# Synthesis and Characterization of 2D Metal-Organic Frameworks for Adsorption of Carbon Dioxide and Hydrogen

**DOI:** 10.3389/fchem.2020.581226

**Published:** 2020-11-05

**Authors:** Piwai Tshuma, Banothile C. E. Makhubela, Christophe A. Ndamyabera, Susan A. Bourne, Gift Mehlana

**Affiliations:** ^1^Department of Chemical Technology, Faculty of Science and Technology, Midlands State University, Gweru, Zimbabwe; ^2^Center for Synthesis and Catalysis Department of Chemical Sciences, Faculty of Science, University of Johannesburg, Johannesburg, South Africa; ^3^Department of Chemistry, Faculty of Science, University of Cape Town, Cape Town, South Africa

**Keywords:** carbon dioxide, topology, metal-organic framework, adsorption, catalysis

## Abstract

The reaction of Cd(NO_3_)_2_·4H_2_O and Zn(NO_3_)_2_·6H_2_O with the bipyridyl dicarboxylate ligand H_2_bpydc (2,2′-bipyridine-4,4′-dicarboxylic acid) afforded two porous metal organic frameworks [Cd(bpydc)_2_(DMF)_2_·2DMF]_n_ (JMS-3) and [Zn(bpydc)(DMF)·DMF]_n_ (JMS-4). X-ray diffraction studies revealed that both JMS-3 and JMS-4 crystallize in the monoclinic crystal. The MOFs possess 2D interdigited networks with (**sql**) topology. Sorption studies showed that the activated phase of JMS-3 had CO_2_ volumetric uptakes of 26.50 and 30.89 cm^3^ (STP) g^−1^ (1.18 and 1.39 mmol g^−1^) whist JMS-4 gave 10.96 and 16.08 cm^3^ (STP) g^−1^ (0.49 and 0.71 mmol g^−1^) at 298 and 273 K respectively.

## Introduction

One of the most threatening environmental issues is the atmospheric increase in the concentration of CO_2_. Climate change and global warming has been attributed to the gradual increase in CO_2_ in the atmosphere (Poloni et al., 2014; Saeidi et al., 2014; Cuéllar-Franca and Azapagic, 2015; Gunasekar et al., 2016; Hu et al., 2019). Different strategies meant to reduce CO_2_ emissions such as the use of renewable energy and CO_2_ capture and storage (CCS) (Liang et al., 2013) have been proposed and adopted. Although CCS is an attractive method of dealing with CO_2_ emissions, it is of paramount importance to find ways recycling greenhouse gas as well as harnessing renewable sources of energy for industrial applications.

Recent studies have demonstrated that metal-organic frameworks (MOFs) are good candidates for CCS. MOFs are crystalline materials constructed using metal centers and organic linkers (Dhakshinamoorthy et al., 2012; Chughtai et al., 2015). These materials have shown remarkable H_2_ and CO_2_ capture and storage at high pressure. Notably, MOFs have been shown to outperform other materials, such as zeolites, silica and metal oxides, in terms of adsorption capacities for CO_2_ as well as in separation of gases materials (Choi et al., 2009; Li et al., 2011; Anbia and Hoseini, 2012; Ding et al., 2019; Kidanemariam et al., 2019). These high adsorption capacities are attributed to high porosity, large surface area, tunable functionalities as well as uniform-structured cavities (Liu et al., 2014) which makes them promising candidates for diverse applications in gas storage (Yang et al., 2012), separation (Mehlana et al., 2014), catalysis (Valvekens et al., 2013), and sensing (Mehlana et al., 2015a,b; Mehlana et al., 2016). In order to improve the gas adsorption capacity of MOFs toward CO_2_, strategies such as pore size control (Sun et al., 2006; Lin et al., 2011), incorporation of open metal sites (Britt et al., 2009; Liang et al., 2013) grafting of amines into the frameworks and introduction of nitrogen-rich organic linkers (Demessence et al., 2009; Andirova et al., 2016) have been proposed.

As part of our ongoing work to develop novel materials that can be used for carbon dioxide capture and conversion using the bipyridyl dicarboxylate linker, herein, we present two novel MOFs of [Cd_2_(bpydc)_2_(DMF)_2_·2DMF]_n_ and [Zn(bpydc)(DMF)·DMF]_n_ which were successfully synthesized using 2,2′-bipyridine-4,4′-dicarboxylate (bpdc) linker (Tshuma et al., 2020a,b). Topological analysis revealed that the MOFs possess a 2D network with square lattice topology. The MOFs JMS-3 and JMS-4 (where JMS denotes Johannesburg and Midlands State) showed a crystalline to crystalline transformation upon activation. Chemical stability studies of the activated phases revealed that JMS-3a is not stable in a number of solvents while JMS-4a showed greater stability. Both JMS-3a and JMS-4a showed appreciable CO_2_ and H_2_ adsorption capacity. This makes both JMS-3a and JMS-4a potential candidates for CO_2_ storage and conversion.

## Experimental Section

### Materials and Reagents

Cd(NO_3_)_2_·4H_2_O, Zn(NO_3_)_2_·6H_2_O), N,N- dimethylformamide (DMF) and H_2_bpydc were purchased from Sigma-Aldrich and used without further purification unless otherwise mentioned.

### Analytical Methods

Single crystal X-ray diffraction (SCXRD) data collection was done on a Bruker KAPPA APEX II DUO Diffractometer equipped with graphite monochoromated Mo Kα radiation (λ = 0.71073 Å). The XPERT-PRO diffractometer (Cu Kα radiation) was used to collect Powder X-ray diffraction (PXRD) data. A current flow of 40 mA and voltage of 40 kV was used to generate the X-rays. The Perkin Elmer Fourier BX II fitted with an ATR probe was used to collect Fourier Transform Infrared (FITR) spectra of the samples. The thermal profiles of the JMS-3a and JMS-4a were recorded on a TA Discovery Instrument TA-Q50. In a typical experiment, samples weighing between 2 and 5 mg were heated at 10°C min^−1^ within a temperature range of 25–600°C under nitrogen purge gas flow of 50 mLmin^−1^. Adsorption studies were performed using a Micrometrics 3Flex Surface Analyser. The samples were prepared by using a Micrometrics Flowprep with the flow of nitrogen over the samples for 4 h at 60°C. The samples were activated at 150°C under vacuum for 2 h before the analysis to remove all the solvent molecules.

## Synthesis

### Synthesis of [Cd_2_(bpdc)_2_(DMF)_2_·2DMF]_n_ (JMS-3) and [Zn(bpdc)(DMF)·DMF]_n_ (JMS-4)

JMS-3: About 25 mg (0.098 mmol) of H_2_bpydc was predissolved in DMF (10 mL) followed by addition of Cd(NO_3_)_2_·4H_2_O (117 mg, 0.38 mmol). The resulting solution was sealed in a glass vial and heated at 115°C for 8 h to yield colorless block shaped crystals. Elemental Analysis, Found % C 43.3, %H 4, %N 11.4, calculated, % C 43.1, %H 3.99, %N 11.2.

JMS-4: A mixture of H_2_bpydc (27 mg, 0.105 mmol) and Zn(NO_3_)_2_·6H_2_O (112 mg, 0.377 mmol) were dissolved in DMF at room temperature. The reaction mixture was placed in a sealed glass vial and heated at 100°C for 6 h to obtain colorless block crystals. Elemental Analysis, Found % C, 47.5 %H 4.2, %N 12.1, calculated, % C 47.6, %H 4.4, %N 12.3

### Crystallographic Data Collection and Refinement

JMS-3 and JMS-4's single crystal data were collected on a Bruker KAPPA APEX II DUO diffractometer equipped with graphite monochoromated Mo Kα radiation (λ = 0.71073 Å). The SAINT program was used for unit cell refinement and data reduction. The structures of the two MOFs were solved by direct methods (program SHELXS2018) (Sheldrick, 2018) and refined anisotropically on F^2^ full-matrix least-squares with SHELXL (Sheldrick, 2018) using the X-SEED (Barbour, 2001) interface. All non-hydrogen atoms were refined anisotropically The hydrogen atoms were placed in idealized positions using the riding model and assigned temperature factors relative to the parent atom. The crystallographic data for JMS-3 and JMS-4 with CCDC numbers 2010231 and 2010232, respectively, is given in Table 1.

**Table 1:** Crystal data and refinement parameters for JMS-3 and JMS-4.

	**JMS-3**	**JMS-4**
Empirical formula	C_36_H_40_N_8_Cd_2_O_12_	C_18_H_20_N_4_Zn_1_O_6_
Formula weight (gmol^−1^)	1001.58	453.75
Temperature/K	293(2)	150(2)
Crystal system	monoclinic	monoclinic
Space group	P2(1)/c	P2(1)
a/Å	18.4447(13)	9.3824(6)
b/Å	15.0567(11)	14.7232(9)
c/Å	16.0209(11)	14.7089(9)
α/°	90	90
β/°	113.878(1)	101.203(2)
γ/°	90	90
Volume/Å^3^	4068.4(5)	1993.2(2)
Z	4	4
Calculated density (g/cm^3^)	1.635	1.5119
μ(Mo-Kα) /mm^−1^	1.12	1.27
F(000)	2016	936
Crystal size/mm^3^	0.38 × 0.36 × 0.21	0.26 × 0.20 × 0.17
Radiation	MoKα (λ= 0.71073)	MoKα (λ = 0.71073)
2θ Max/°	56.82	52.94
Reflections collected	95569	54023
No. unique data	10205	4105
Goodness of fit on S	1.021	1.085
Final R indexes [I>=2σ (I)]	0.0638	0.0546
Final wR_2_ indexes [all data]	0.0829	0.1052
Largest diff. peak/hole/e Å^−3^	0.69/−0.57	1.15/−0.80

## Results and Discussion

### Structural Description

**Figure 1:**
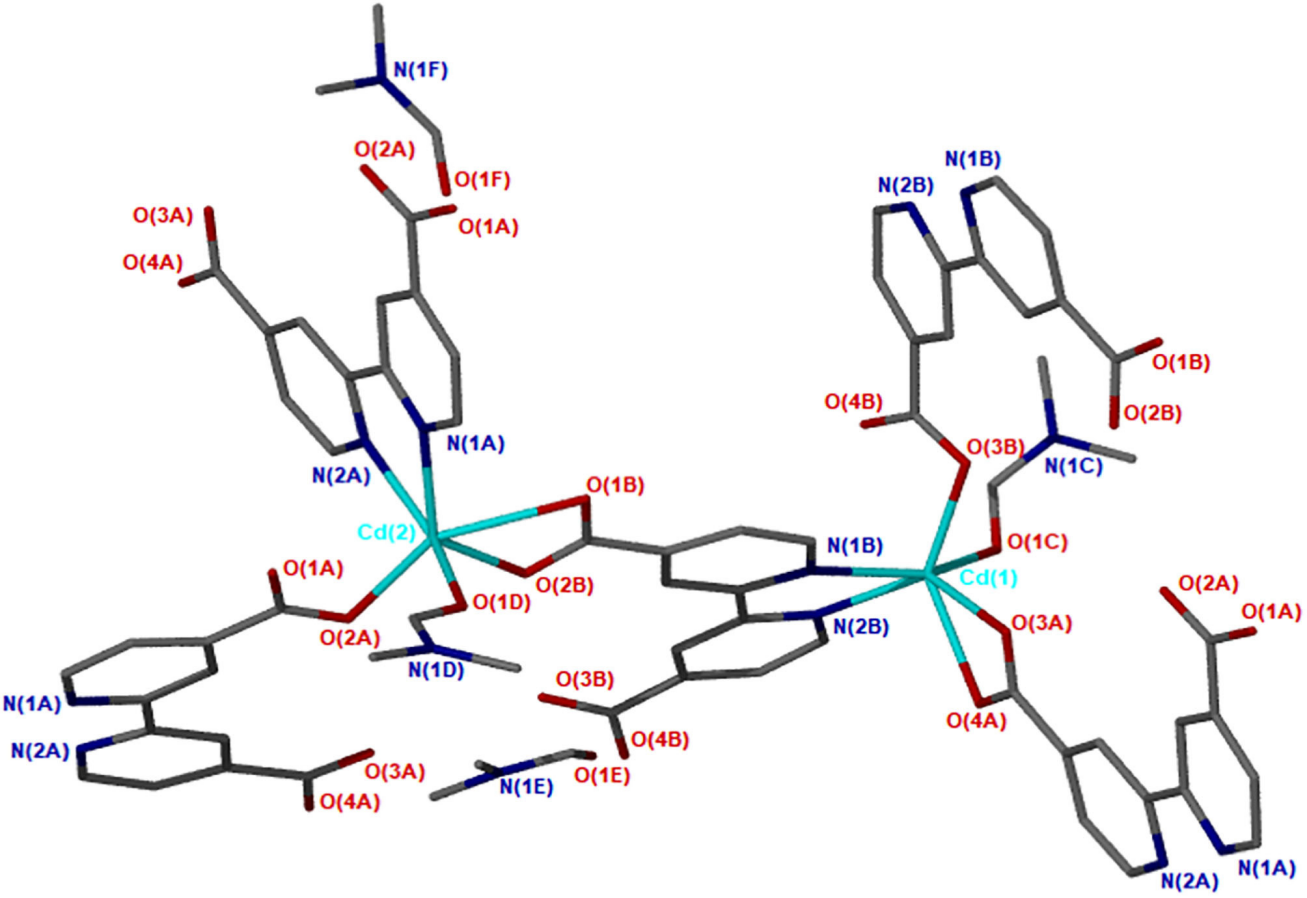
Coordination environment of Cd(II) in JMS-3.

**Figure 2:**
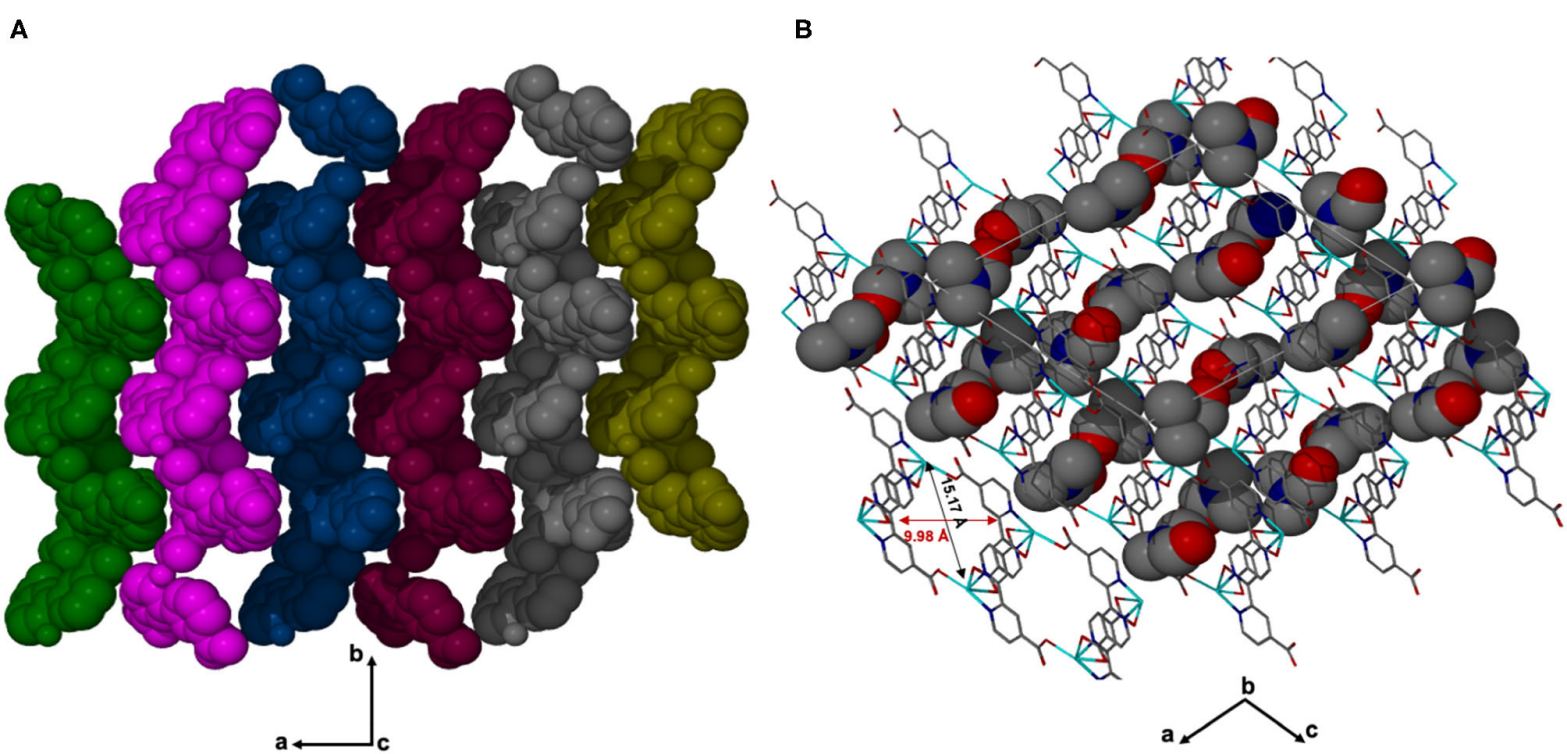
**(A)** Structure of JMS-3 displaying interdigited channels, viewed along *c*-axis. **(B)** The 2D network of JMS-3 guest molecules are drawn with van der Waals radii.

X-ray diffraction analysis using the single crystal method revealed that JMS-3 crystallizes in the monoclinic system and space group P2_1_/c. The asymmetric unit of JMS-3, comprises of two crystallographic independent Cd(II) centers, two deprotonated bpydc linkers, two coordinated DMF molecules and two uncoordinated DMFs. As shown in Figure 1, each cadmium metal center is bound to three bpydc linkers. The carboxylate moiety assumes both bidentate and monodentade binding mode. Two nitrogen atoms of the third linker are chelated to Cd(II). A DMF molecule is also bound to each of the Cd(II) centers. The Cd–N bond length around Cd ranges from 2.318(2) to 2.366 (3) while Cd-O ranges from 2.239 (2) to 2.588(2) Å (see Supplementary Table 1). The bond length around the two metal centers are different. This explains why the structure contains two crystallographically independent Cd(II) centers. The Cd(II) metal center assumes a distorted octahedral geometry with bond angles ranging from 54.19(8) to 152.57(9) and 84.89(8) to 154.73(8)° for Cd1 and Cd2, respectively. The packing diagram of JMS-3 displays 2D networks which are interdigited (Figure 2A). Coordinated and uncoordinated DMF molecules are trapped in the rectangular channels as illustrated in Figure 2B. Weak intermolecular and intramolecular C-H…O bonding exist between the host framework and guest molecules. These are suggested to reinforce the geometry and impart stability in the MOF. Guest molecules occupy 53.8% of the unit cell volume, as estimated in PLATON (Spek, 2009).

**Figure 3:**
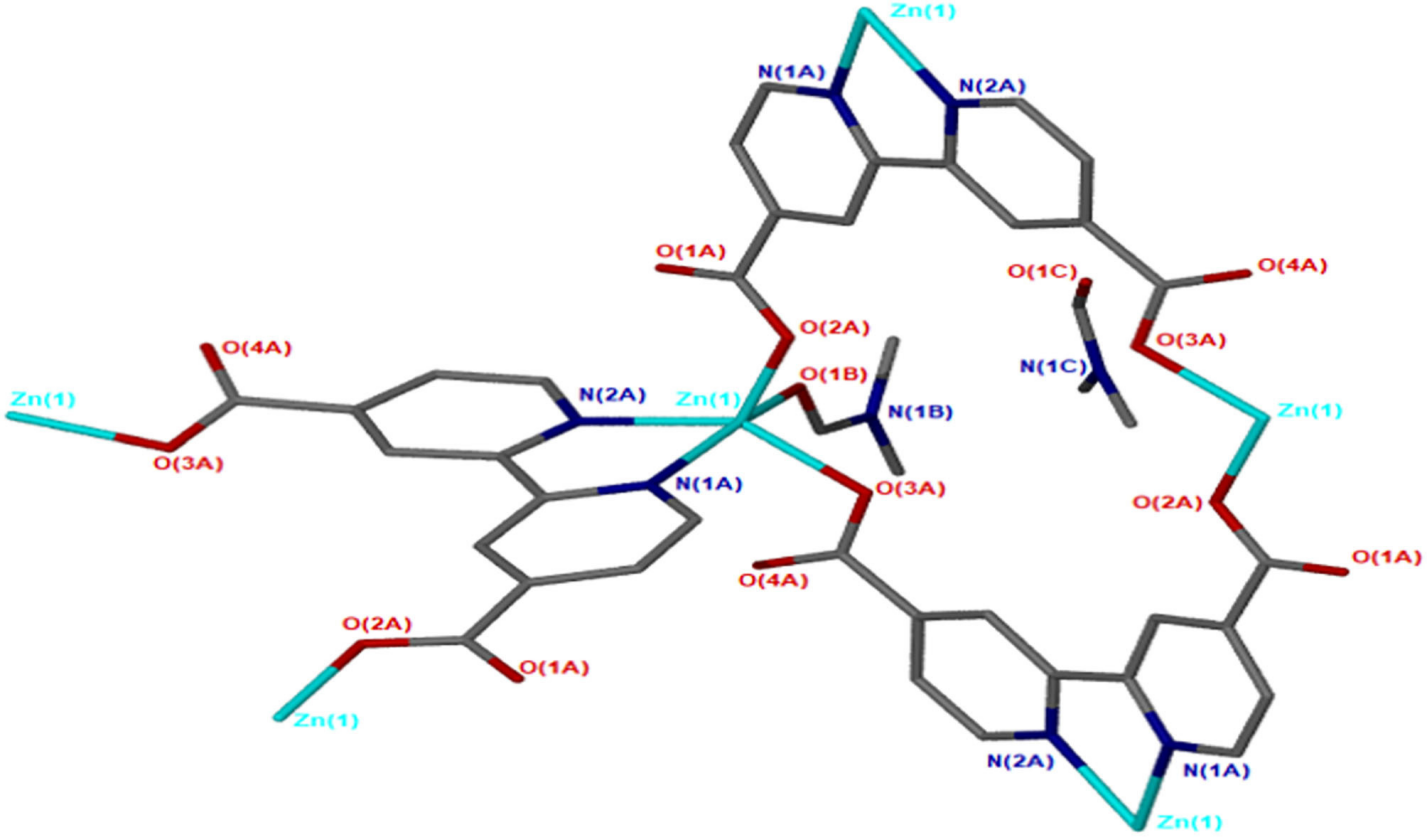
Coordination geometry of Zn(II) in JMS-4.

**Figure 4:**
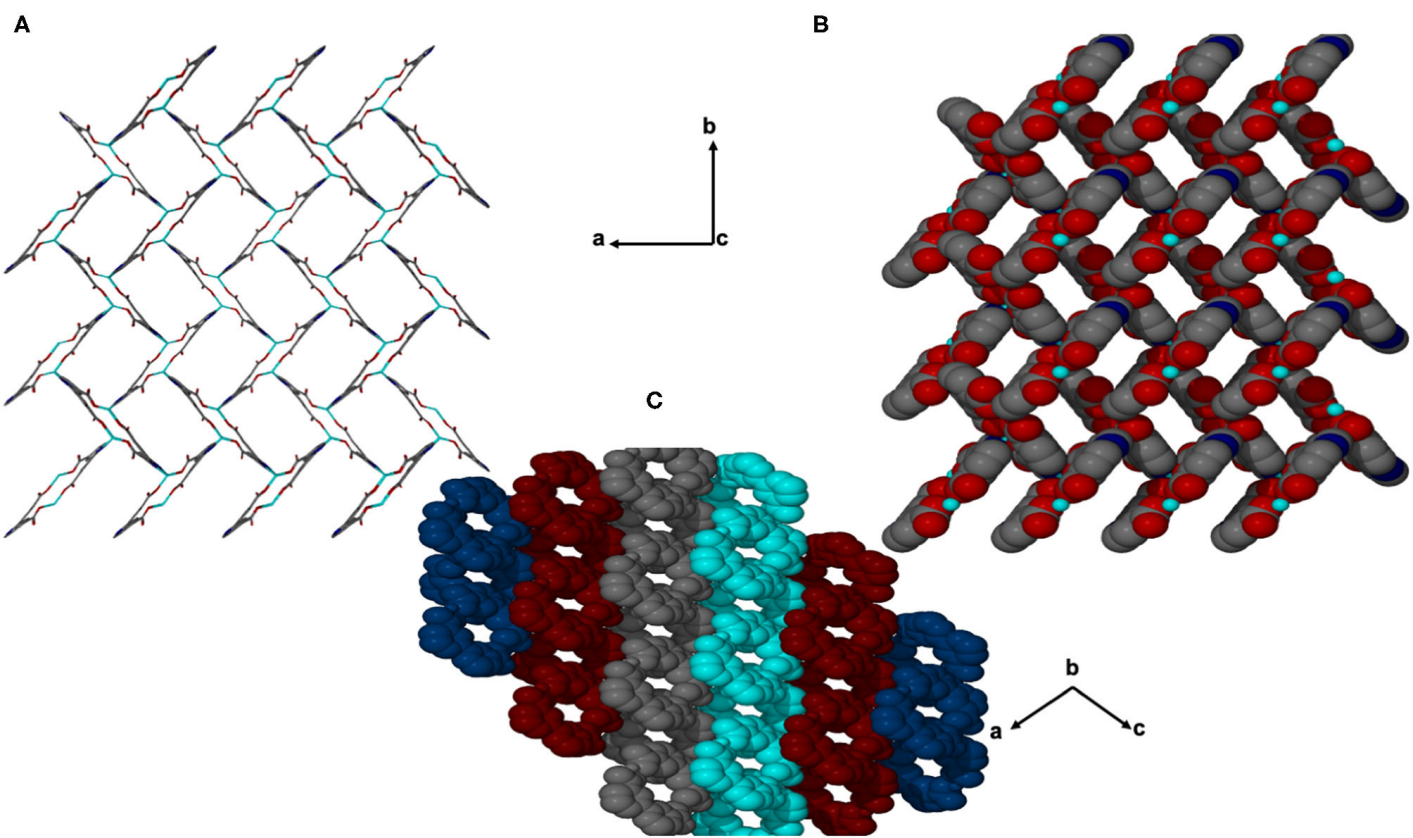
Channels of JMS-4 **(A)** stick form, **(B)** space filling viewed along c axis, and **(C)** Packing diagram drawn in Van der waals radii displaying the interdigited chains.

JMS-4 crystallizes in the P2_1_ space group. The asymmetric unit comprises of one zinc metal center, one deprotonated bpydc linker and two DMF molecules. The Zn(II) is coordinated to one bpydc linker through two monodentate Zn(II)-O bonds to the carboxylate moiety, two bipyridyl nitrogen atoms in a chelating fashion and one DMF molecule. This gives rise to a trigonal bipyramidal coordination as illustrated in Figure 3. The Zn–N ranges from 2.1019(2) to 2.177(3) Å while the Zn-O bond length ranges from and 1.976(2) to 2.104(2), respectively. These bond lengths are given in Supplementary Table 2 and are consistent with literature reports. Analysis of the bond angles around the zinc center shows that they are within a range of 76.40(10) to 159.71(10)°. Figure 4 shows the packing diagram of JMS-4 with 2D layers packing in a similar fashion to that of JMS-3. The observed rectangular channels which are occupied by both coordinated and uncoordinated DMF molecules (10.342 × 9.036 Å) run along *b*-axis. PLATON (Spek, 2009) estimates that the volume occupied by both the coordinated and uncoordinated DMF molecules accounts for 52.3% of the unit cell volume. There are no noticeable interactions between the 2D network and the uncoordinated guest DMF molecules. However, weak intermolecular hydrogen bonding between the 2D layers and π…π interactions of the aromatic rings stabilizes the structure.

### Thermal Analysis and PXRD Studies

**Figure 5:**
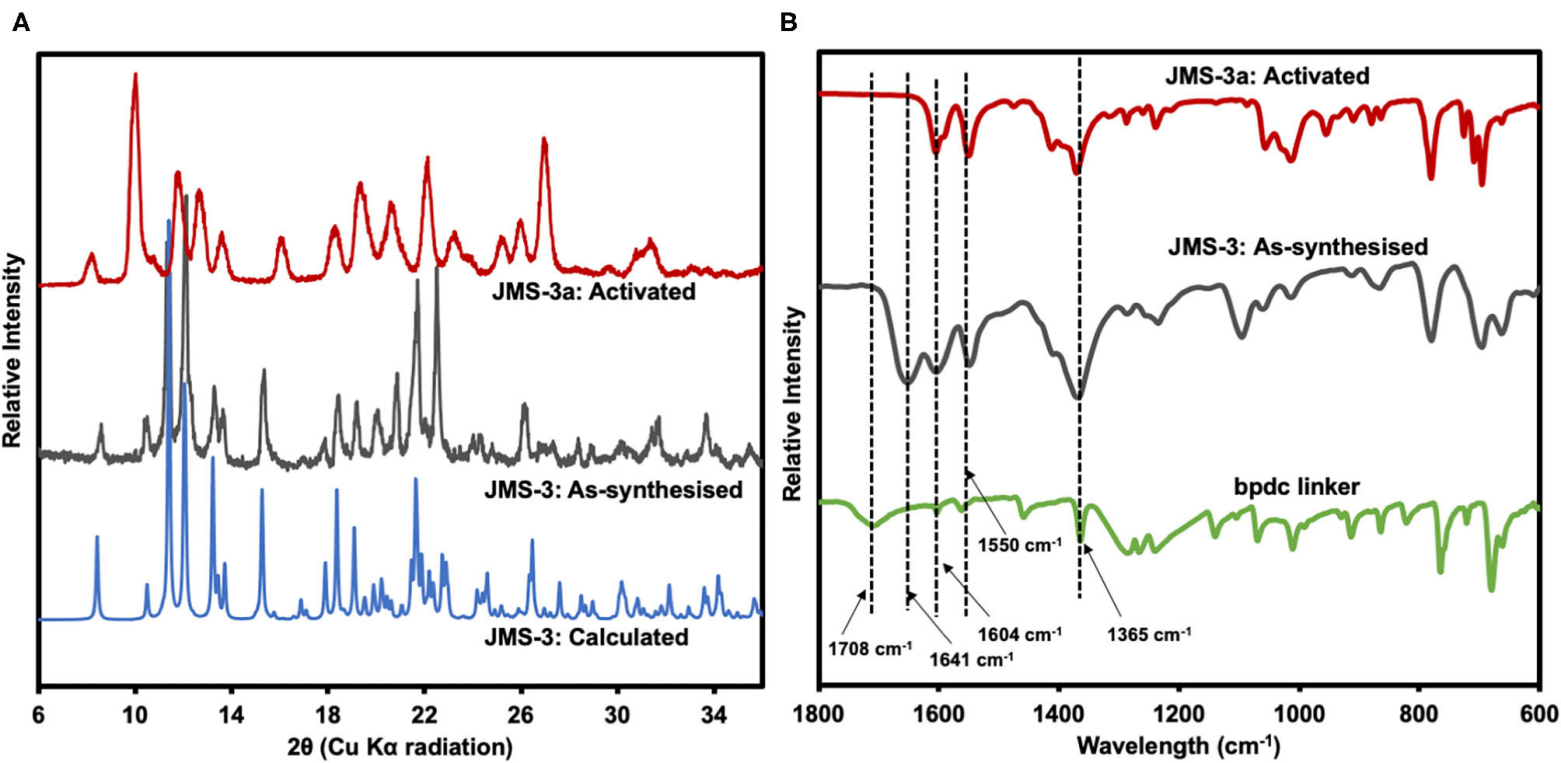
**(A)** PXRD patterns of calculated, as-synthesized and activated JMS-3. **(B)** IR spectra of the linker, as synthesized and activated JMS-3.

Thermal analysis of JMS-3 by TGA illustrated in Supplementary Figure 1 exhibits an initial mass loss of 28.6% between 100 and 150°C. The observed weight loss is attributed to the extrusion of four DMF molecules (calculated 29.2%). The MOF was activated by replacing the DMF molecules by methanol followed by heating at 24 h at 80°C to give JMS-3a. As shown in Figure 5A, a comparison of the experimental PXRD of JMS-3 with the calculated pattern, reveals a good match confirming phase purity of the as-synthesized MOF. Upon removal of the guest DMF molecules, JMS-3 undergoes a structural transformation as evidenced by the appearance of new diffraction peaks around 10, 16, and 27 two-theta positions.

FTIR results presented in Figure 5B shows that the band at 1708 cm^−1^ in the linker shifts to 1,604 cm^−1^ in JMS-3, which is evidence that the carboxylate moiety is coordinated to the Cd(II) center. These results are consistent with single crystal X-ray data. Successful activation of the MOF is evidenced by the disappearance of the FTIR band at 1,641 cm^−1^, which is attributed to the carbonyl stretch of DMF molecules. The band located at 1,550 cm^−1^ is attributed to the C-C of the benzene ring. The carboxylate asymmetric and symmetric carboxylate stretches were observed at 1,604 and 1,365 cm^−1^, respectively, with a magnitude of separation of 239 cm^−1^. After activation of JMS-3, the position of the bands does not change suggesting that the integrity of the carboxylate functional group is retained (Lu et al., 2016).

The thermal behavior of JMS-4 was examined by TGA. An initial weight loss of 32.5% observed in the TGA curve in Supplementary Figure 2 is attributed to the loss of two DMF molecules modeled in the asymmetric unit (calculated 32.2%). Framework decomposition occurs around 350°C. No significant weight loss occurs below 350°C on the TGA curve of the activated framework (JMS-4a), indicating solvent molecules have been completely removed, although due to humidity, about 4% loss was still observed on the activated sample curve.

**Figure 6:**
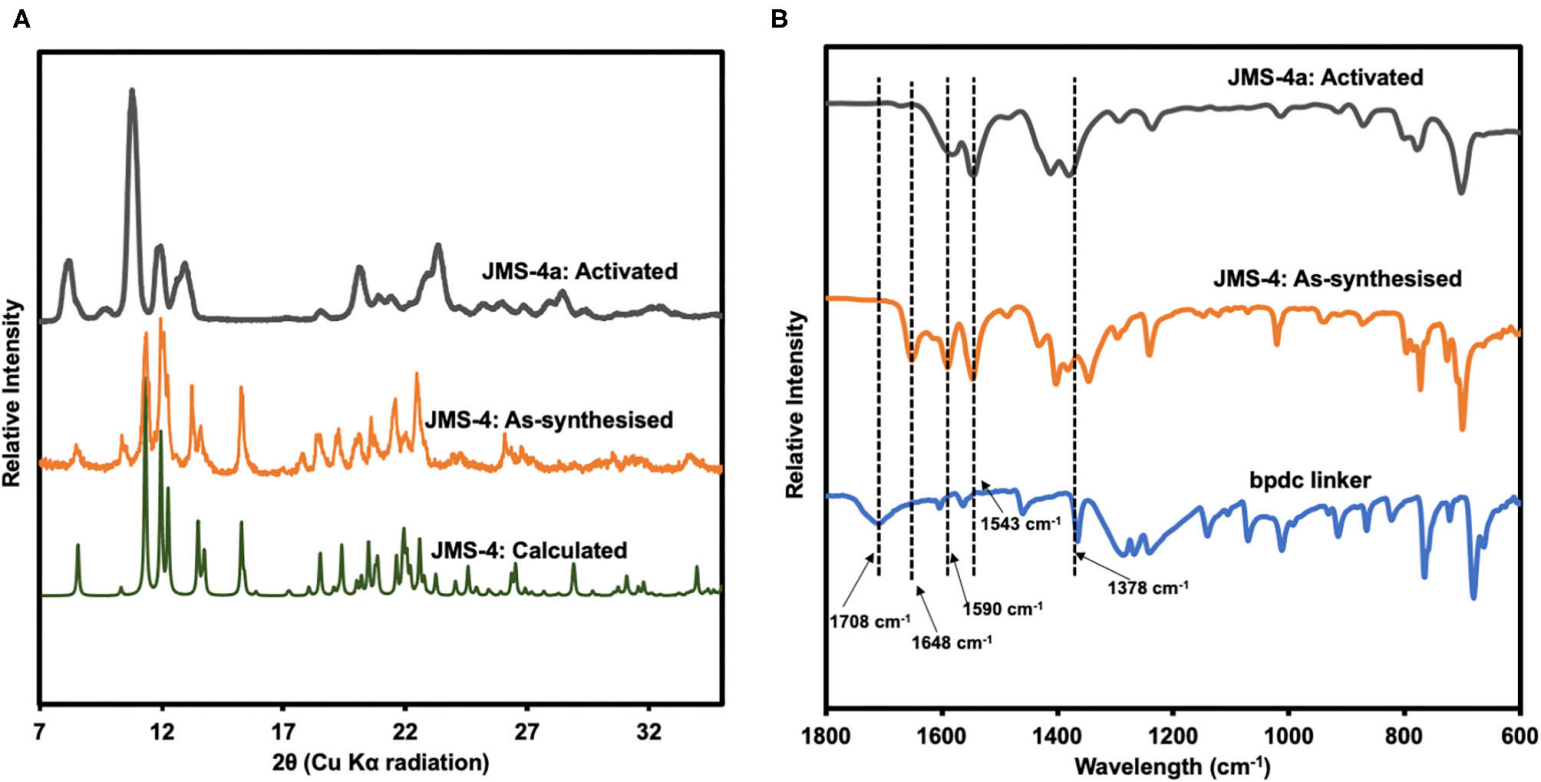
**(A)** PXRD patterns of calculated, experimental and activated JMS-4, **(B)** IR spectrum of the linker, as-synthesized and activated JMS-4.

PXRD studies confirmed the phase purity of JMS-4 as evidenced by excellent agreement between the calculated and experimental diffraction patterns (Figure 6A). Upon activation of JMS-4 a new phase is obtained. The IR spectrum of as-synthesized JMS-4 (Figure 6B) shows a band at 1,648 cm^−1^ assignable to carbonyl stretch of DMF molecules. This band disappears upon activation. The bands at 1,590 and 1,378 cm^−1^ are assigned to the asymmetric and symmetric stretches of the carboxylate group respectively, with a magnitude of separation of 212 cm^−1^. Upon activation, the FTIR analysis suggests that the integrity of carboxylate moiety with respect to its binding mode is not altered (Lu et al., 2016).

### Topological Analysis

**Figure 7:**
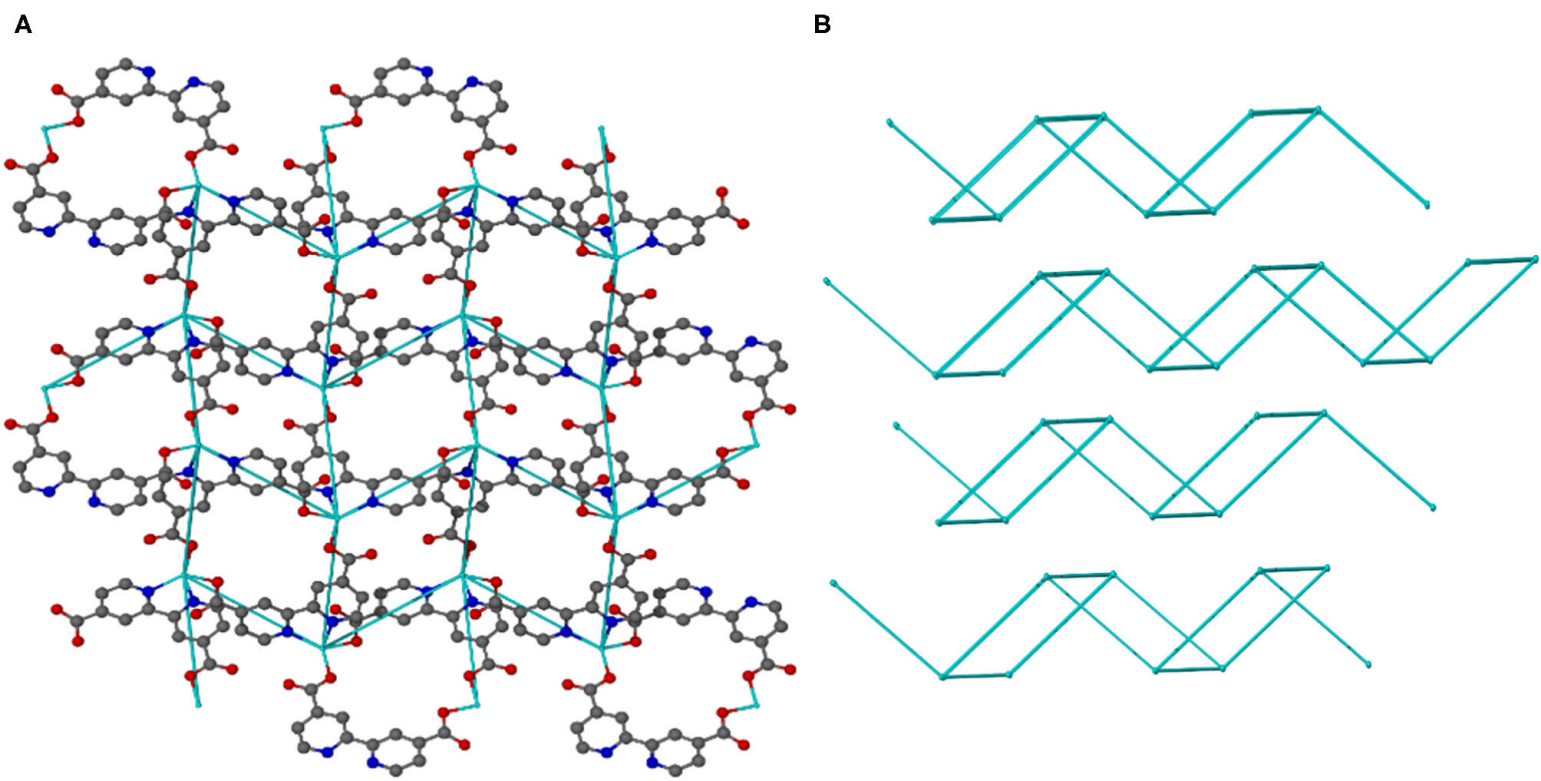
**(A)** 4-connected 2D net of JMS-3 and **(B)** Zig-zag packing in JMS-3.

Topological analysis of JMS-3 and JMS-4 was perfomed using Topos computer programme (Coelho, 2007). As shown in Figure 7A, the compound was reduced to a simple net by considering each metal center as a 4 connected node. Each cadmium center is connected to the other cadimium center by the linear bpydc bridges to produce a square-lattice (**sql**) topology (Mehlana et al., 2013, 2017). The packing in Figure 7B is a zig-zag 2D layered network. The packing is in an offset position with approximately equi-distances between the layers (Mehlana et al., 2015a; Tella et al., 2017). Although the two MOFs have diffrenent geometries around the metal center and crystallize in different space groups, they exhibit the same **sql** topology.

### Non-isothermal Desorption Kinetics

The kinetics of DMF desorption in JMS-3 and JMS-4 were investigated to determine the activation energy required to remove the guest molecules. The Ozawa method, which relies on the use of several heating rates and the temperature being recorded at specific conversion levels for each heating rate was used (Ozawa, 1965). The method made makes use of the equation below:

$$\begin{array}{l} \text{log βα}=\text{ log}\left(\frac{A\alpha .\text{Eax}}{g\left(a\right)R}\right)-2.315-0.457\left(\frac{Eaa}{RTa}\right), \end{array}$$

Where β_α_ – heating rate

A_α_ – frequency factor

E_aα_ – activation energy

T_a_ – temperature at each conversion level

g_α_ – kinetic model

**Figure 8:**
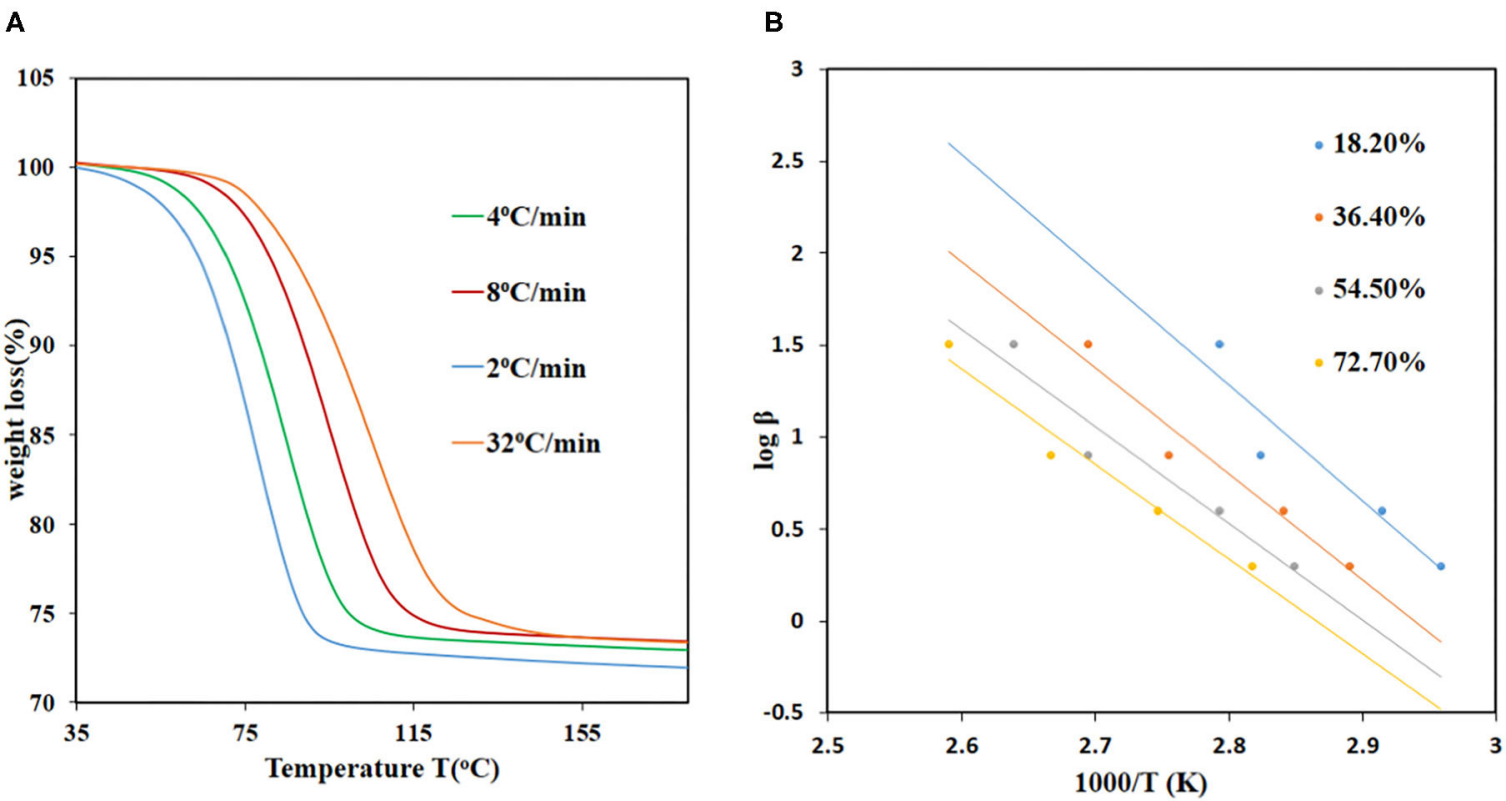
Desorption kinetics for JMS-4: **(A)** TGA traces obtained using different heating rates as a function of temperature, **(B)** log β vs. 1/T, the gradient of the slope were used to calculate the activation energies.

The equation can be used to calculate the activation energies at each conversion level. The TGA data was converted to extent-of-reaction (α) vs. temperature and the information obtained from the curves used to plot graphs of log β_α_ vs. 1/T_a_. The activation energies for JMS-3 ranged from 68.9 to 90.1 kJ mol^−1^ within a conversion range 16.7–83.3% conversion levels, respectively, while the activation energies ranging from 68.9 to 91.6 kJ/mol for JMS-4 within a conversion range of 22–80%. Figure 8A shows the desorption kinetic profiles of JMS-4 at different heating rates from the non-isothermal runs while Figure 8B depicts plots of log β vs, 1/T, the slope of which is ensued the activation energies at the different conversion levels. The kinetic profiles of JMS-3 is presented in Supplementary Figure 3. The two MOFs exhibited similar average activation energies of 81.65 and 82.35 kJ/mol for JMS-3 and JMS-4, respectively. The observed energies are consistent with the modeled DMF molecules being coordinated to the metal center and the structural features of the MOFs.

### Chemical Stability Studies

**Figure 9:**
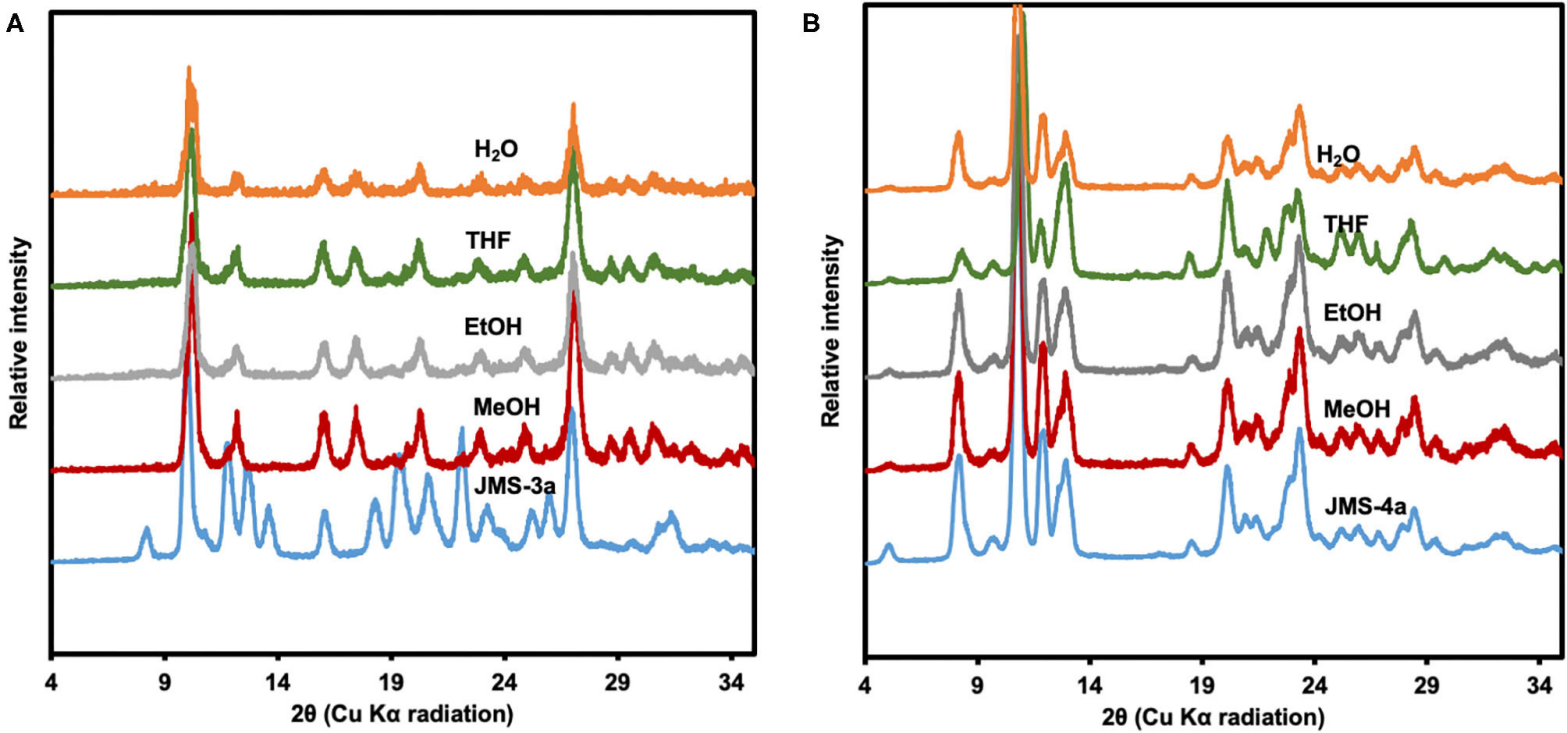
**(A)** PXRD patterns of JMS-3a **(B)** JMS-4 soaked in several solvents for 36 h.

To test the chemical stability, the activated MOFs, **JMS-3a** and **JMS-4a** were soaked in different organic solvents for 36 h. As shown in the PXRD pattern of the recovered samples (Figure 9A), **JMS-3a** is not stable under different chemical environments. A new phase, which is uniform throughout is obtained. Contrary to **JMS-3a**, the structure of **JMS-4a** is maintained as shown by an excellent agreement between the PXRD paterrns of the activated phase and the ones obtained after soaking the materials in different solvents for 36 h (Figure 9B).

### Carbon Dioxide and Hydrogen Adsorption Studies

**Figure 10:**
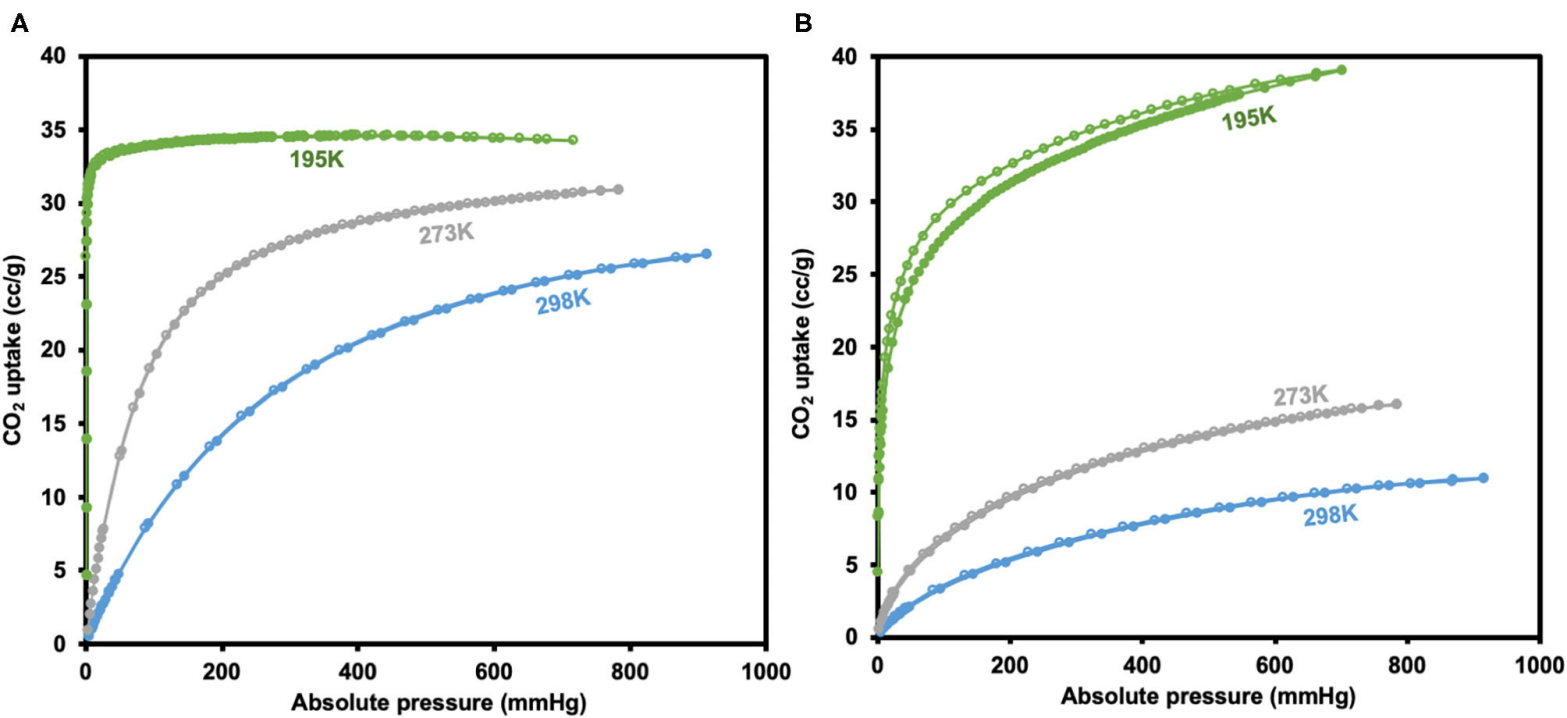
CO_2_ adsorption-desorption isotherms for JMS-3a **(A)** and JMS-4a **(B)** (closed circles represent adsorption and open circles desorption).

CO_2_ adsorption isotherms of JMS-3a and MOF JMS-4a carried at temperatures (298, 273, and 195 K) and 1 atm pressure (Figure 10) revealed a Type-1 isotherm, which is typical of microporous materials (Alhamami et al., 2014). JMS-3a gave volumetric uptakes of 26.50 and 30.89 cm^3^ (STP) g^−1^ (1.18 and 1.39 mmolg^−1^) whist JMS-4a gave 10.96 and 16.08 cm^3^ (STP) g^−1^ (0.49 and 0.71 mmolg^−1^) at 298 and 273 K, respectively. At 195 K, the MOFs show a rapid uptake of CO_2_ at low pressure with volumetric uptake of 34.66 and 38.84 cm^3^ (STP)g^−1^ (1.52 and 1.74 mmolg^−1^) for JMS-3a and JMS-4a, respectively, confirming inherent permant porosity of the MOFs. CO_2_ sorption studies at 273 K revealed a surface area of 151 and 36 m^2^g^−1^ for JMS-3a and JMS-4a, respectively. The use of CO_2_ sorption for BET calculation is controversial, however, Bae et al. demonstrated that this method maybe applied to ultramicroporous materals at 273 K (Kim et al., 2016). JMS-3a perform exceptionally well for CO_2_ capture at 273 and 298 K, probably due to its high suface area compared to JMS-4a. Upon activation, the PXRD pattern of JMS-3a (Figure 3) shows that structural transformation is not much as compared to JMS-4a (Figure 7). The adsorption branches of the isotherm carried out at 273 and 298 K were used to obtain a precise prediction over the quantity of CO_2_ adsorbed at the saturation point (Ugale et al., 2018). The estimated values of isosteric heat (Q_st_) of adsorption for JMS-3a were found to be 33–34 kJ mol^−1^ at loading values ranging from 0.12 to 0.99 mmol g^−1^(Supplementary Figure 4). CO_2_ isotherms for JMS-4a between 273 and 298 K revealed isosteric heats of adsorption values of 29–31 kJ mol^−1^ at loading values of 0.32–0.45 mmol g^−1^ (Supplementary Figure 5). The observed CO_2_ uptake indicate moderate to strong interaction of CO_2_ with the MOFs and are comparable to other MOFs reported in literature as illustrated in Table 2.

**Table 2:** A comparison of CO_2_ adsorption capacity for various Cd and Zn based MOFs at 1 atm.

**MOF material**	**Temp (K)**	**CO_**2**_ uptake (mmolg^**−1**^)**	**Qst (kJmol^**−1**^)**	**References**
[{Cd_2_(L-glu)_2_(bpe)}]_n_	273 298	0.92 0.64	40.8	Ugale et al., 2017
[Cd_2_(Tzc)_2_]n	195	2.45	-	Zhong et al., 2011
[Zn(TCPB)_2_]_n_	195 273	6.29 2.89	-	Lin et al., 2012
[Zn(hfipbb)(bpt)]_n_	195	1.71	35.8	Chatterjee and Oliver, 2018
	273 298	0.96 0.62		
[Cd_2_(bpdc)_2_]_n_	195	1.52	34.4	This work
	273 298	1.39 1.18		
[Zn(bpdc)]_n_	195	1.74	30.7	This work
	273 298	0.71 0.49		

*L-glu, L-glutamate dianion; bpe, 1.2-bis(4-pyridyl)ethylene; Tzc, tetraazolate-5-carboxylate; TCPB, 1,3,5-tri(4-carboxyphenoxy)benzene; hfipbb, 4,4′- (hexafluoroisooropylidene)bis (benzoate) and bpt, 4-amino-3,5-bis(4-pyridyl)-1,2,4-triazole. Reports in this table include adsorption capacities in mmolg^-1^. (Some have been converted to these units from the originally reported units)*.

**Figure 11:**
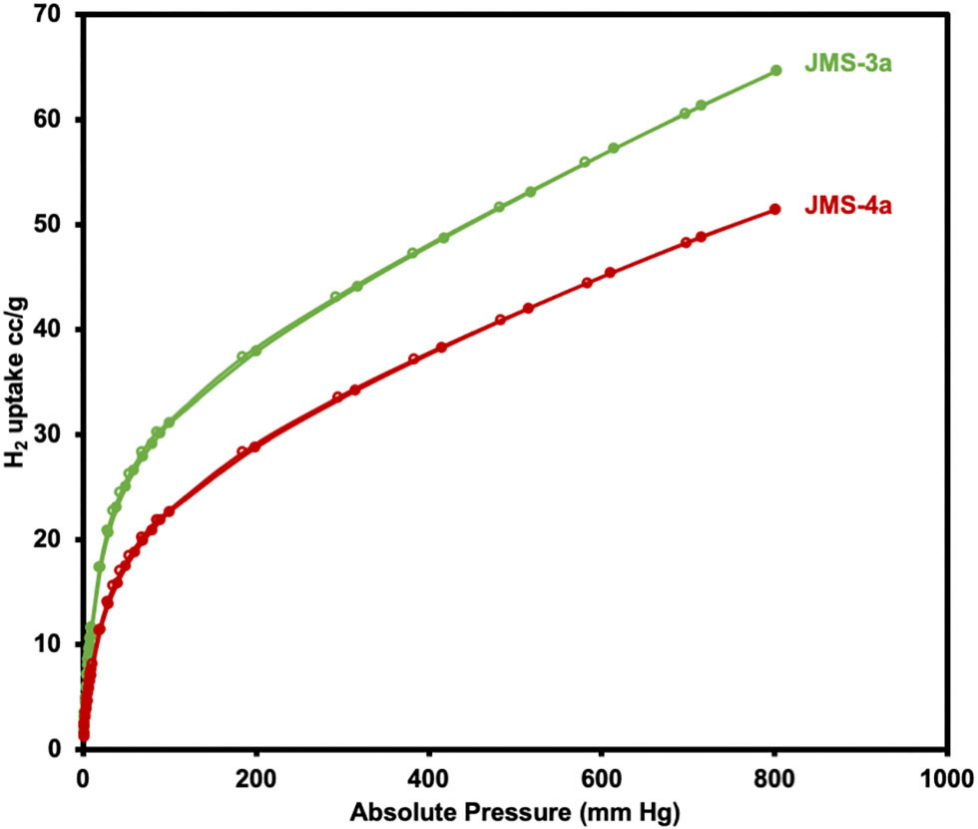
Adsorption and desorption isotherms of JMS-3a and JMS-4a at 77K for H_2_ studies.

Hydrogen sorption studies in Figure 11 reveals a storage capacity of up to 65 and 51 cm^3^g^−1^ for JMS-3a and JMS-4a at 800 mmHg and 77K, corresponding to sorption of 0.58 and 0.46 wt.% (2.88 and 2.29 mmolg^−1^), and displays a significant hysteresis loop. The hydrogen adsorption values are comparable to that of [Cd_2_(Tzc)_2_]n (0.55 wt.%) and [Zn(hfipbb)(bpt)]_n_ (0.44 wt.%) but less than that of [Zn(TCPB)2]_n_ (0.80 wt.%) under similar conditions.

## Conclusions

The structures of two MOFs JMS-3 and JMS-4 were reported. These MOFs were analyzed by diffraction and spectroscopic methods. Although the two MOFs crystallized in different space groups, topological analysis revealed that they both exhibit an **sql** net. Desorption kinetic studies for the removal of the DMF molecules revealed that the activation energy required in both systems was comparable (an average of 79 kJ mol^−1^ for JMS-3 and 81 kJ mol^−1^ for JMS-4). The activated phases JMS-3a and JMS-4a were exposed to ethanol, methanol, water and THF to test their chemical stability. As opposed to JMS-3a, JMS-4a did not reveal any structural changes when soaked in these solvents that are normally employed in the catalytic hydrogenation of CO_2_. This makes JMS-4a a potential candidate for application in CO_2_ conversion reactions. Future work will focus on encapsulating molecular catalysts in JMS-4a for CO_2_ hydrogenation studies.

## Data Availability Statement

The datasets presented in this study can be found in online repositories. The names of the repository/repositories accession number(s) can be found in the article/Supplementary Material.

## Author Contributions

PT and CN carried out the experimental work. BM, SB, and GM supervised the experimental work. The first draft of the manuscript was written by PT and was then revised by all the co-authors.

## Conflict of Interest

The authors declare that the research was conducted in the absence of any commercial or financial relationships that could be construed as a potential conflict of interest.

## References

[B1] Alhamami M. Doan H. Cheng C. H. (2014). A review on breathing behaviors of metal-organic-frameworks (MOFs) for gas adsorption. Materials 7, 3198–3250. 10.3390/ma7043198 28788614 PMC5453333

[B2] Anbia M. Hoseini V. (2012). Development of MWCNT@MIL-101 hybrid composite with enhanced adsorption capacity for carbon dioxide. Chem. Eng. J. 191, 326–330. 10.1016/j.cej.2012.03.025

[B3] Andirova D. Cogswell C. F. Lei Y. Choi S. (2016). Effect of the structural constituents of metal organic frameworks on carbon dioxide capture. Microporous Mesoporous Mater. 219, 276–305. 10.1016/j.micromeso.2015.07.029

[B4] Barbour L. J. (2001). X-Seed - a software tool for supramolecular crystallography. J. Supramolecul. Chem. 1, 189–191. 10.1016/S1472-7862(02)00030-8

[B5] Britt D. Furukawa H. Wang B. Glover T. G. Yaghi O. M. (2009). Highly efficient separation of carbon dioxide by a metal-organic framework replete with open metal sites. Proc. Natl. Acad. Sci. U.S.A. 106, 20637–20640. 10.1073/pnas.0909718106 19948967 PMC2791636

[B6] Chatterjee N. Oliver C. L. (2018). A dynamic, breathing, water-stable, partially fluorinated, two-periodic, mixed-ligand Zn(II) metal-organic framework modulated by solvent exchange showing a large change in cavity size: gas and vapor sorption studies. Crystal Growth Design 18, 7570–7578. 10.1021/acs.cgd.8b01391

[B7] Choi S. Drese J. H. Jones C. W. (2009). Adsorbent materials for carbon dioxide capture from large anthropogenic point sources. ChemSusChem 2, 796–854. 10.1002/cssc.200900036 19731282

[B8] Chughtai A. H. Ahmad N. Younus H. Laypkov A. Verpoort F. (2015). Metal-organic frameworks: versatile heterogeneous catalysts for efficient catalytic organic transformations. Chem. Soc. Rev. 44, 6804–6849. 10.1002/chin.201546250 25958955

[B9] Coelho A. (2007). TOPAS-Academic, Version 4.1 (Computer Software), Coelho Software, Brisbane.27409075

[B10] Cuéllar-Franca R. M. Azapagic A. (2015). Carbon capture, storage and utilisation technologies: a critical analysis and comparison of their life cycle environmental impacts. J. CO2 Utilization 9, 82–102. 10.1016/j.jcou.2014.12.001

[B11] Demessence A. Alessandro D. M. D. Foo M. L. Long J. R. (2009). Strong CO_2_ binding in water-stable, triazole-bridged metal-organic framework functionalised with ethylenediamine. J. Am. Chem. Soc. 131, 8784–8786. 10.1021/ja903411w 19505094

[B12] Dhakshinamoorthy A. Alvaro M. Garcia H. (2012). Commercial metal-organic frameworks as heterogeneous catalysts. Chem. Commun. 48, 11275–11288. 10.1039/c2cc34329k 23044896

[B13] Ding M. Flaig R. W. Jiang H. L. Yaghi O. M. (2019). Carbon capture and conversion using metal-organic frameworks and MOF-based materials. Chem. Soc. Rev. 48, 2783–2828. 10.1039/C8CS00829A 31032507

[B14] Gunasekar G. H. Park K. Jung K. D. Yoon S. (2016). Recent developments in the catalytic hydrogenation of CO_2_ to formic acid/formate using heterogeneous catalysts. Inorganic Chem. Front. 3, 882–895. 10.1039/C5QI00231A

[B15] Hu Z. Wang Y. Shah B. B. Zhao D. (2019). CO_2_ capture in metal-organic framework adsorbents: an engineering perspective. Adv. Sustain. Syst. 3:1800080 10.1002/adsu.201800080

[B16] Kidanemariam A. Lee J. Park J. (2019). Recent innovation of metal-organic frameworks for carbon dioxide photocatalytic reduction. Polymers 11:2090. 10.3390/polym11122090 31847223 PMC6960843

[B17] Kim K. C. Yoon T. U. Bae Y. S. (2016). Applicability of using CO_2_ adsorption isotherms to determine BET surface areas of microporous materials. Microporous Mesoporous Mater. 224, 294–301. 10.1016/j.micromeso.2016.01.003

[B18] Li J. R. Ma Y. Colin McCarthy M. Sculley J. Yu J. Jeong H. K. (2011). Carbon dioxide capture-related gas adsorption and separation in metal-organic frameworks. Coordination Chem. Rev. 255, 1791–1823. 10.1016/j.ccr.2011.02.012

[B19] Liang Z. Du J. Sun L. Xu J. Mu Y. Li Y.. (2013). Design and synthesis of two porous metal–organic frameworks with *Nbo* and *Agw* topologies showing high CO_2_ adsorption capacity. Inorganic Chem. 52, 10720–10722. 10.1021/ic4017189 24041328

[B20] Lin X. M. Li T. T. Wang Y. W. Zhang L. Su C. Y. (2012). Two ZnIImetal-organic frameworks with coordinatively unsaturated metal sites: structures, adsorption, and catalysis. Chem. J. 7, 2796–2804. 10.1002/asia.201200601 23038041

[B21] Lin Z. J. Liu T. F. Xu B. Han L. W. Huang Y. B. Cao R. (2011). Pore-size tuning in double-pillared metal-organic frameworks containing cadmium clusters. Cryst. Eng. Comm. 13, 3321–3324. 10.1039/c1ce05099k

[B22] Liu J. Chen L. Cui H. Zhang J. Zhang L. Su C. Y. (2014). Applications of metal-organic frameworks in heterogeneous supramolecular catalysis. Chem. Soc. Rev. 6011–6061. 10.1039/C4CS00094C 24871268

[B23] Lu S. M. Wang Z. Li J. Xiao J. Li C. (2016). Base-free hydrogenation of CO_2_ to formic acid in water with an iridium complex bearing a: N, N ′-Diimine Ligand.” Green Chem. 18, 4553–4558. 10.1039/C6GC00856A

[B24] Mehlana G. Bourne S. A. Ramon G. (2014). The role of C–H…π interactions in modulating the breathing amplitude of a 2D square lattice net: alcohol sorption studies. CrystEngComm 16:8160 10.1039/C4CE00496E

[B25] Mehlana G. Bourne S. A. Ramon G. Öhrström L. (2013). Concomitant Metal Organic Frameworks of Cobalt(II) and 3-(4-Pyridyl) benzoate: optimized synthetic conditions of solvatochromic and thermochromic systems. Crystal Growth Design 13, 633–644. 10.1021/cg301312v

[B26] Mehlana G. Chitsa V. Mugadza T. (2015a). Recent advances in metal–organic frameworks based on pyridylbenzoate ligands: properties and applications. RSC Adv. 5, 88218–88233. 10.1039/C5RA15575D

[B27] Mehlana G. Ramon G. Bourne S. A. (2016). A 4-fold interpenetrated diamondoid metal-organic framework with large channels exhibiting solvent sorption properties and high iodine capture. Micropor. Mesoporous Mater. 231, 21–30. 10.1016/j.micromeso.2016.05.016

[B28] Mehlana G. Wilkinson C. Dzesse C. N. T. Ramon G. Bourne S. A. (2017). Structural diversity observed in two-dimensional square lattice metal-organic frameworks assembled from Zn(II) and 3-(4-Pyridyl)Benzoate. Crystal Growth Design 17, 6445–6454. 10.1021/acs.cgd.7b01101

[B29] Mehlana G. Wilkinson C. Ramon G. Bourne S. (2015b). Reversible thermochromic and mechanochromic behaviour in a 3D hydrogen bonded discrete complex. Polyhedron 98, 224–229. 10.1016/j.poly.2015.06.016

[B30] Ozawa T. (1965). A new method of analyzing thermogravimetric data. Chem. Soc. Jpn. 1881–1886. 10.1246/bcsj.38.1881

[B31] Poloni R. Lee K. Berger R. F. Smit B. Neaton J. B. (2014). Understanding trends in CO_2_ adsorption in metal-organic frameworks with open-metal sites J. Phys. Chem. Lett. 5, 861–865. 10.1021/jz500202x 26274079

[B32] Saeidi S. Amin N. A. S. Rahimpour M. R. (2014). Hydrogenation of CO2 to value-added products - a review and potential future developments. J. CO_2_ Utilization 5, 66–81. 10.1016/j.jcou.2013.12.005

[B33] Sheldrick G. M. (2018). Crystal structure refinement with SHELXL. Acta Crystallogr. Section C 71, 3–8. 25567568 10.1107/S2053229614024218 PMC4294323

[B34] Spek A. L. (2009). Structure validation in chemical crystallography. Acta Crystallographica 65(Pt 2), 148–155. 10.1107/S090744490804362X 19171970 PMC2631630

[B35] Sun D. Ma S. Ke Y. Collins D. J. Zhou H. C. (2006). An interweaving MOF with high hydrogen uptake. J. Am. Chem. Soc. 128, 3896–3897. 10.1021/ja058777l 16551082

[B36] Tella A. C. Mehlana G. Alimi L. O. Bourne S. A. (2017). Solvent-free synthesis, characterization and solvent-vapor interaction of Zinc(II) and copper(II) coordination polymers containing nitrogen-donor ligands. Zeitschrift Fur Anorganische Und Allgemeine Chem. 643, 523–530. 10.1002/zaac.201600460

[B37] Tshuma P. Makhubela B. C. E. Bingwa N. Mehlana G. (2020a). Palladium(II) immobilized on metal-organic frameworks for catalytic conversion of carbon dioxide to formate. Inorganic Chem. 59, 6717–6728. 10.1021/acs.inorgchem.9b03654 32330382

[B38] Tshuma P. Makhubela B. C. E. Öhrström L. Bourne S. A. Chatterjee N. Beas I. N. (2020b). Cyclometalation of Lanthanum(Iii) based MOF for catalytic hydrogenation of carbon dioxide to formate. RSC Adv. 10, 3593–3605. 10.1039/C9RA09938G PMC9048731 35497735

[B39] Ugale B. Dhankhar S. S. Nagaraja C. M. (2017). Construction of 3D homochiral metal-organic frameworks (MOFs) of Cd(II): selective CO_2_ adsorption and catalytic properties for the knoevenagel and henry reaction. Inorganic Chem. Front. 4, 348–359. 10.1039/C6QI00506C

[B40] Ugale B. Singh Dhankhar S. Nagaraja C. M. (2018). Exceptionally stable and 20-connected lanthanide metal-organic frameworks for selective CO_2_ capture and conversion at atmospheric pressure. Crystal Growth Design 18, 2432–2440. 10.1021/acs.cgd.8b00065

[B41] Valvekens P. Vermoortele F. De Vos D. (2013). Metal–organic frameworks as catalysts: the role of metal active sites. Catal. Sci. Tech. 3:1435 10.1039/c3cy20813c

[B42] Yang Q. Guillerm V. Ragon F. Wiersum A. D. Llewellyn P. L. Zhong C.. (2012). CH_4_ storage and CO_2_ capture in highly porous zirconium oxide based metal–organic frameworks. Chem. Commun. 48, 9831–9833. 10.1039/c2cc34714h 22932495

[B43] Zhong D. C. Zhang W. X. Cao F. L. Jiang L. Lu T. B. (2011). A three-dimensional microporous metal-organic framework with large hydrogen sorption hysteresis. Chem. Commun. 47, 1204–1246. 10.1039/C0CC03506H 21107465

