# Supplementary material for: Synthesis and Characterization of 2D Metal-Organic Frameworks for Adsorption of Carbon Dioxide and Hydrogen
Source: Front Chem. 2020 Nov 5;8:581226. doi: 10.3389/fchem.2020.581226 (PMC7674654; doi:10.3389/fchem.2020.581226)
Supplement: Supplementary file 1 [file Data_Sheet_1.docx]

**Supporting information for**

**Synthesis and Characterization of 2D Metal-Organic Frameworks for adsorption of carbon dioxide and hydrogen**

Piwai Tshuma^1^, Banothile CE Makhubela^2*^, Christophe A. Ndamyabera^3^, Susan A Bourne^3^ and Gift Mehlana^1*^

^1^Department of Chemical Technology, Faculty of Science and Technology, Midlands State University, Gweru, Zimbabwe

^2^ University of Johannesburg, Center for Synthesis and Catalysis Department of Chemical Sciences, Faculty of Science, Kingsway Campus: C2 Lab 328, Auckland Park, 2006, South Africa

^3^ University of Cape Town, Department of Chemistry, Faculty of Science, PD Hahn Building 7701 Rondebosch, Cape Town, South Africa

**Table S1:** Bond distances around Cd(II) in **JMS-3**.

| **Bond Type** | **Bond Length (Å)** | **Bond Type** | **Bond Length (Å)** |
| --- | --- | --- | --- |
| Cd1-O3B | 2.239(2) | Cd2-O2A | 2.252(2) |
| Cd1-O1C | 2.299(2) | Cd2-O2B | 2.275(2) |
| Cd1-N2B | 2.318(2) | Cd2-O1D | 2.281(2) |
| Cd1-O3A | 2.322(3) | Cd2-N1A | 2.327(2) |
| Cd1-N1B | 2.335(3) | Cd2-N2A | 2.366(2) |


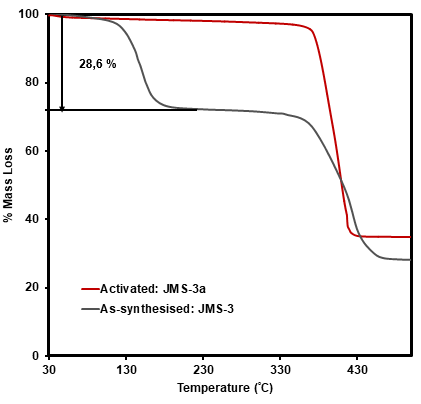


**Figure S1:** TGA traces of experimental and activated **JMS-3**.

**Table S2:** Bond distances around Zn(II) in **JMS-4**.

| **Bond Type** | **Bond Length (Å)** | **Bond Type** | **Bond Length (Å)** |
| --- | --- | --- | --- |
| Zn1-O2A | 1.976(2) | Zn1-N2A | 2.101(2) |
| Zn1-O3A | 2.022(2) | Zn1-N1A | 2.177(3) |
| Zn1-O1B | 2.104(2) |  |  |


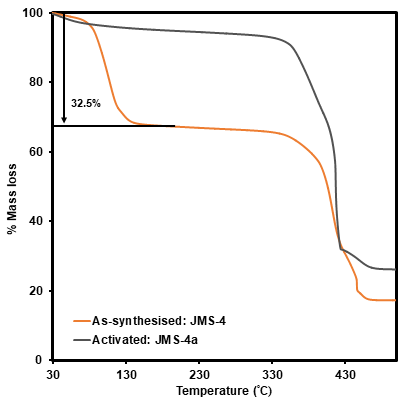


**Figure S2:** TGA traces of experimental and activated **JMS-4**.


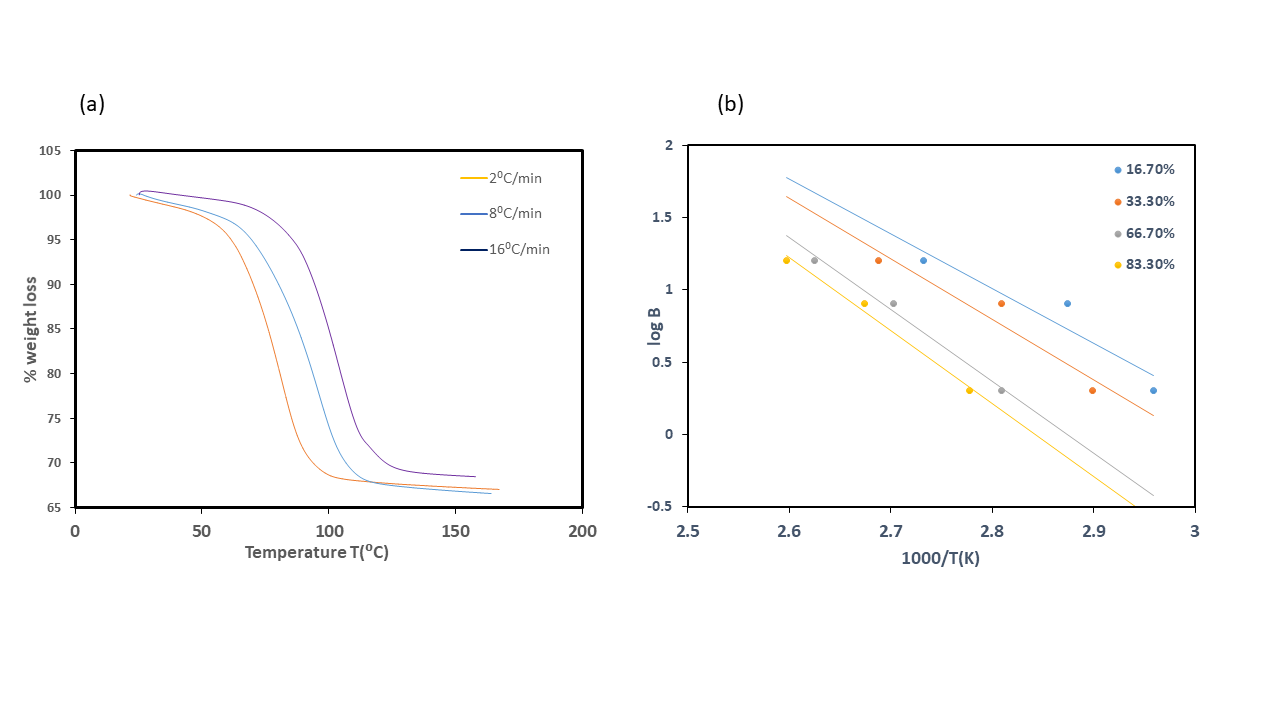


**Figure S3**: Kinetic desorption profiles of JMS-3 (left) and a plot of log B versus 1000/T for JMS-4


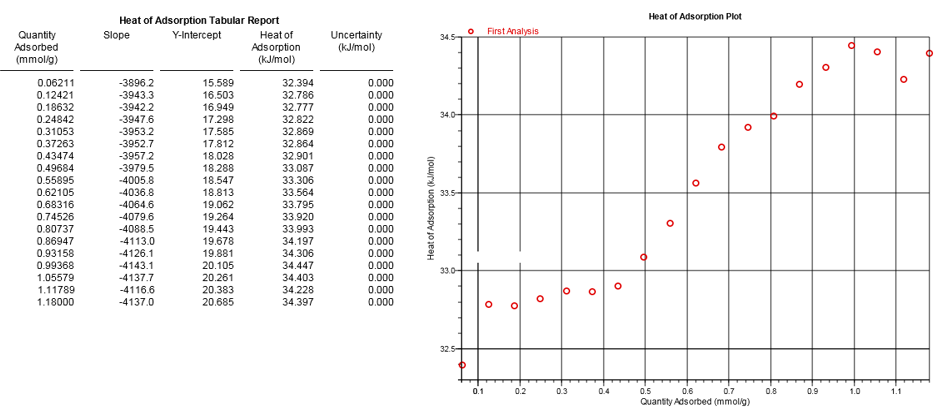


**Figure S4:** Isosteric heat of adsorption for **JMS-3a**


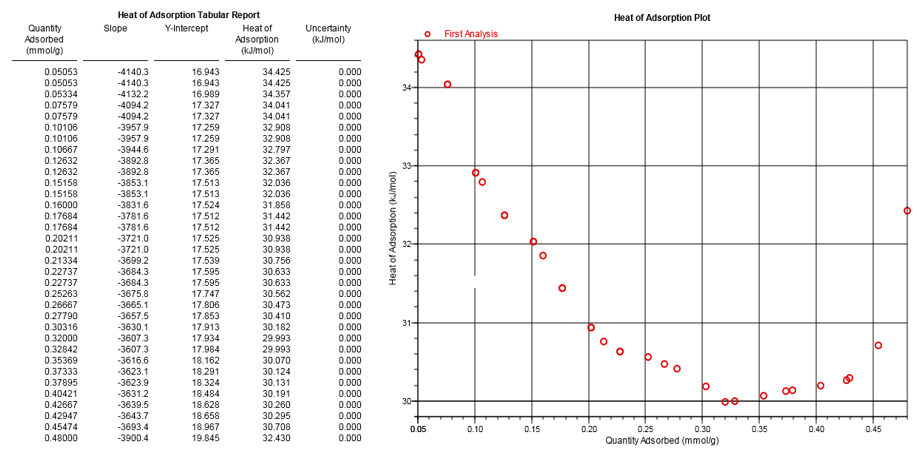


**Figure S5:** Isosteric heat of adsorption for **JMS-4a**
